# The Special Issue on Neonatology for the 10th Anniversary of *Children*: From Preclinical Findings to Bringing Families to the Centre of Contemporary Neonatal Care

**DOI:** 10.3390/children12050622

**Published:** 2025-05-12

**Authors:** Karel Allegaert

**Affiliations:** 1 Department of Pharmaceutical and Pharmacological Sciences, KU Leuven, 3000 Leuven, Belgium; karel.allegaert@kuleuven.be; 2 Department of Development and Regeneration, KU Leuven, 3000 Leuven, Belgium; 3 Department of Hospital Pharmacy, Erasmus MC, 3015 GD Rotterdam, The Netherlands

## 1. Introduction

Neonatology is a specialized branch of medicine, focussed on the care of newborns, either related to preterm birth or other medical conditions like sepsis, asphyxia, or congenital malformations. In the 1960s, specialized neonatal intensive care units (NICU) emerged, transforming the care landscape for newborns, informed by other disciplines, and evolving from eminence-based practices to more evidence-based approaches [1].

Weighted to other disciplines, this means that our discipline is still rather young; therefore, there is still much to investigate, as reflected in the diversity of topics covered in the current Special Issue [1]. In addition, the creation of NICUs also resulted in a more robust ecosystem, with a higher degree of confidence and opportunities to shift to the treatment of neonates with more complex care demands, like micro-preemies, or complex cardiac malformations or genetic syndromes. Treatment modalities changed, from delivery room management to care bundle concepts (respiratory, neurological, nutritional, pharmacological, and family-centred care), as have the outcomes of preterm neonates or neonates with specific conditions [2,3]. Related to prevention, screening and treatment have evolved significantly over the last decade, improving outcomes. Prevention and screening strategies have been developed and implemented for prenatal screening, dry blood spots, cardiac screening, and hearing screening [4].

This diversity is also reflected in the topics covered in this Special Issue, ranging from preclinical (murine model, human milk fat composition) and epidemiology (impact of race on outcome, impact of fetal growth restriction on behavioural outcome), to guidelines (oxygenation monitoring, hypoglycemia) and several papers on family-centred care and parent-related impact (impact of family-centred and developmental care on outcome, relevance of the family context, post-discharge impact of parent support), ending with two papers on screening-related practices (mother’s knowledge on dried blood spot screening, usefulness of polymorphism screening for neonatal bilirubinaemia management).

## 2. Overview of the Published Articles

### 2.1. Pre-Clinical

In a preclinical murine bronchopulmonary dysplasia model (BPD), Chen et al. observed that neonatal hyperoxia exposure caused an arrest of lung development, as well as an obstacle to the myelination process in the white matter of the immature brain, with a decline in myeline basic protein in the generation period of myelin and persistent astrogliosis (contribution 1). This informs us that BPD is a systemic disease, not limited to the pulmonary system.

The impact of human milk fatty acid composition on growth velocity in preterm infants was reported by Ahmed et al. In essence, and based on 15 mother–infant dyads, the growth velocity increased with the decrement in C16 and increment in C20:2n6, while the lipid profile of preterm human milk was found to be low for some essential fatty acids, which may affect the quality of preterm infants’ nutrition (contribution 2). Interestingly, there is a link between both findings, as the lipid profile is also crucial to ensure myelination.

### 2.2. Epidemiology

The progress mentioned in the introduction is most clearly reflected in the data on mortality. Qattea et al. hereby described that there has been a significant decrease in mortality in black and white populations in the last two decades in the United States (contribution 3). However, when stratifying the population by many significant epidemiologic and hospital factors, the mortality remained consistently higher in black populations throughout the study years.

In a Spanish cohort of 70 former fetal growth-restriction cases, behavioural performance was assessed at the age of 6 years by Benitez Marin et al. Higher behavioural disability rates were observed, providing additional evidence on the negative relationship between the birth weight percentile and the total behavioural scale score (contribution 4). Both papers inform us on the clinical relevance of specific covariates that determine short- or long-term outcome of former NICU graduates.

### 2.3. Guideline Adherence

European guidelines recommend the use of pulse oximetry (PO) during newborn resuscitation, especially when there is a need for positive pressure ventilation or supplemental oxygen. Kolstad et al. therefore evaluated these practices based on video recordings in 230 ventilated newborns at delivery. In total, 97% of resuscitated newborns had PO applied, in line with resuscitation guidelines. However, the proportion of time with a useful PO signal during ventilation and during the first 10 minutes on the resuscitation table was only 5% and 35%, respectively (contribution 5).

Neonatal hypoglycemia remains an issue of debate as it is a preventable cause of brain injury and neurodevelopmental impairment. Unfortunately, Giouleka et al. had to conclude that—after a comprehensive review on the guidelines—there is still no clear definition, nor consistent treatment policy. Thus, the establishment of specific diagnostic criteria and uniform protocols for the management of this common biochemical disorder is of paramount importance as it may allow early identification of infants at risk and the establishment of effective preventive measures or treatments to improvement outcomes (contribution 6).

Both papers are quite illustrative of the logistics related to and the complexity of either implementing what is already known (the pulse oximetry measurement during neonatal ventilation), while the paper on neonatal hypoglycemia reinforms us on the many known unknows in this field.

### 2.4. Parents Matter, Bringing Families to the Centre of Contemporary Neonatal Care

Based on a pre-post design in 200 high-risk preterm neonates with family-centred care and developmental care in the intervention group, Alsadaan et al. reported that family centred care and developmental care in the intervention group were associated with improved cognitive, motor, and language scores, as well as with a shorter length of stay (contribution 7).

In a qualitative descriptive study on parent and NICU clinician experiences and perspectives, Dahan et al. concluded that their study highlights how the quality of care is positively impacted by clinicians’ appreciation of the family context and the complex relationship between a large multidisciplinary interprofessional team and the family in an intensive care unit, while also highlighting the difficulties in its practical application (contribution 8).

Post discharge, Munoz et al. observed that parent support (supportive presence and quality of assistance during a complex problem-solving task) had the greatest impact on high-birth risk (≤27 gestational weeks) toddler brain development (frontal lobe grey matter volume, emotion regulation); thus, early parent interventions may normalize preterm child neurodevelopment and have lasting impacts (contribution 9).

All papers clearly illustrate and stress that families should be at the centre of contemporary neonatal care, and that this is not limited to during the neonatal stay but clearly extends to the impact and outcome after discharge.

### 2.5. Screening/Prevention

Di Gangi et al. explored what mothers know about newborn bloodspot screening and the source they use to acquire this knowledge in a Flemish representative sample of 200 subjects and hereby concluded that the consent practices and knowledge level was reasonably good, the information leaflets were perceived to be supporting, while key health care providers were midwives and nurses (contribution 10).

In a well conducted study, Riskin et al. reported that both the genetics of glucose-6-phosphate-dehydrogenase (G6PD) and Uridine Diphosphate Glucuronosyl Transferase 1A1 (UGT1A1) were associated with higher risks of developing clinically relevant neonatal hyperbilirubinemia. The results of this study highlight the possibility for future implementation of molecular genetic screening to identify infants at specific increased risk for significant neonatal hyperbilirubinemia (contribution 11).

A lot of new ideas or concepts have emerged in the field of neonatal newborn bloodspot screening, including a shift towards whole genome screening concepts. The paper on bilirubin-related polymorphisms illustrates one potential useful application, while Di Gangi informs us that we should not forget effective communication with parents and healthcare providers to ensure appropriate consent practices, knowledge level, and overall confidence in these practices.

## 3. Conclusions

In conclusion, the diversity of topics and aspects covered reflect the diversity of research lines needed to make progress, unveil knowledge gaps, share practices, and shift further towards a more evidence-based approach to ensure further improvements in outcomes.
